# N-Doped CrS_2_ Monolayer as a Highly-Efficient Catalyst for Oxygen Reduction Reaction: A Computational Study

**DOI:** 10.3390/nano12173012

**Published:** 2022-08-30

**Authors:** Zengming Qin, Zhongxu Wang, Xiaofeng Li, Qinghai Cai, Fengyu Li, Jingxiang Zhao

**Affiliations:** 1 Key Laboratory for Photonic and Electronic Bandgap Materials, Ministry of Education, School of Physics and Electronic Engineering, Harbin Normal University, No. 1, Shida Street, Harbin 150025, China; 2 College of Chemistry and Chemical Engineering, Harbin Normal University, Harbin 150025, China; 3 School of Physical Science and Technology, Inner Mongolia University, Hohhot 010021, China

**Keywords:** CrS_2_ monolayer, non-metal doping, oxygen reduction reaction, overpotential, density functional theory

## Abstract

Searching for low-cost and highly-efficient oxygen reduction reaction (ORR) catalysts is crucial to the large-scale application of fuel cells. Herein, by means of density functional theory (DFT) computations, we proposed a new class of ORR catalysts by doping the CrS_2_ monolayer with non-metal atoms (X@CrS_2_, X = B, C, N, O, Si, P, Cl, As, Se, and Br). Our results revealed that most of the X@CrS_2_ candidates exhibit negative formation energy and large binding energy, thus ensuring their high stability and offering great promise for experimental synthesis. Moreover, based on the computed free energy profiles, we predicted that N@CrS_2_ exhibits the best ORR catalytic activity among all considered candidates due to its lowest overpotential (0.41 V), which is even lower than that of the state-of-the-art Pt catalyst (0.45 V). Remarkably, the excellent catalytic performance of N@CrS_2_ for ORR can be ascribed to its optimal binding strength with the oxygenated intermediates, according to the computed linear scaling relationships and volcano plot, which can be well verified by the analysis of the p-band center as well as the charge transfer between oxygenated species and catalysts. Therefore, by carefully modulating the incorporated non-metal dopants, the CrS_2_ monolayer can be utilized as a promising ORR catalyst, which may offer a new strategy to further develop eligible electrocatalysts in fuel cells.

## 1. Introduction

The rising energy demand and depletion of fossil fuels have attracted increasing interest in alternative energy sources. This is because the traditional fossil fuels are not renewable and will produce harmful combustion products, such as CO, CO_2_, NO, and SO_2_, which thus pose a serious challenge to human health and environmental protection [1,2,3,4,5,6]. In this regard, electrochemical energy storage and conversion technologies, such as fuel cells, were regarded as efficient and clean devices for reducing the global energy shortage [7,8]. However, the efficiency of these energy-related devices was greatly determined by the oxygen reduction reaction (ORR) due to its sluggish kinetics and high overpotential [9,10,11,12,13]. At present, Pt-based materials represent the most promising ORR catalysts [14,15,16,17]. However, their practical application is severely limited by their high price and low abundance. Thus, the search for highly efficient, low-cost alternative electrocatalysts is urgent to boost the ORR.

In recent years, single-layer transition metal dichalcogenides (TMDs) have become a hotspot of theoretical and experimental research owing to their large surface area, stability, and peculiar electronic structure [18,19,20,21,22,23]. Thus, TMD-based materials have been widely employed in nanoelectronics, nanophotonics, the absorber layer in solar cells, anode materials, field-effect transistors, and so on [24,25,26,27,28]. More interestingly, the heteroatom doped TMDs have attracted great interest because they have more active sites, low cost, high stability, and high efficiency as potential electrocatalytic catalysts [29,30,31,32,33,34,35]. For example, Xiong et al. reported that co-doped MoS_2_ possesses high catalytic activity for oxygen evolution reaction [29]. Moreover, Lv et al. proposed that N-doped MoS_2_ displayed a high faradaic efficiency and low onset overpotential for CO_2_ electroreduction to CO [30]. Theoretically, Li et al. demonstrated that B-doping causes the VS_2_ monolayer to exhibit outstanding catalytic activity towards nitrogen reduction reactions [31]. Moreover, Singh et al. reported that the ORR activity of N-doped WS_2_ monolayers should originate from the introduction of spin density caused by N doping [32]. In addition, Tian et al. showed that Co doped 1T-TiS_2_ exhibits significantly enhanced performance toward the ORR [33].

As a representative of 2D TMDs, the chromium disulfide (CrS_2_) monolayer was synthesized via the chemical vapor deposition (CVD) method in 2019 [21]. Interestingly, due to its unique properties, the CrS_2_ monolayer has wide applications in spintronic devices [36,37]. For example, Chen et al. suggested that CrS_2_ has the most diverse electronic and magnetic properties: antiferromagnetic (AFM) metallic, non-magnetic (NM) semiconductor, and ferromagnetic (FM) semiconductor with a Curie temperature of ~1000 K [36]. Moreover, Zhang et al. reported that the magnetic properties of CrC_2_ monolayer can be effectively tuned by doping metal atoms [37].

Inspired by the interesting properties of the CrS_2_ monolayer, in this work, we explored the potential of several non-metal-doped CrS_2_ monolayers (X@CrS_2_, X = B, C, N, O, Si, P, Cl, As, Se, and Br) as ORR catalysts by performing comprehensive DFT computations. According to the computed formation energies and binding energies of these X@CrS_2_ systems, we suggested that these doped CrS_2_ candidates are very likely to be synthesized in experiments and possess extremely high stability. Moreover, the N@CrS_2_ catalyst was screened out as an eligible ORR catalyst with a rather low overpotential of 0.41 V, which originates from its optimal binding strengths with oxygenated intermediates based on the linear scaling relationships and volcano plot.

## 2. Materials and Methods

All computations were performed based on spin-polarized density functional theory, as implemented in the Vienna ab initio simulation package (VASP 541) [38,39]. The projector augmented wave (PAW) was adopted to describe the interactions between ions and electrons [40,41]. The generalized gradient approximation (GGA) in the form of Perdew–Burke–Ernzerhof (PBE) was adopted for the exchange-correlation functional [42]. Notably, the PBE functional is, nowadays, the most commonly used functional for solid-state calculations, which remains the best for the solids containing 3d-transition elements [43,44,45]. In particular, our purpose in the present work is to search for the ideal ORR catalysts among various candidates, and we thus mainly focus on the catalytic tendency of these candidates for ORR. Thus, although some more modern and better functionals have been proposed in recent years [46,47], the identified scaling relations of oxygenated species on various electrocatalysts and the derived conclusions (such as the corresponding catalytic activity) will not change. The DFT+D3 method in the Grimme scheme was employed to describe the possible weak interactions of the oxygenated species and the catalysts [48]. An energy cutoff of 500 eV was used for the plane wave (Table S1), and the convergence criteria of force and energy were set to 0.02 eV Å^−1^ and 10^−5^ eV, respectively. A k-point of 3 × 3 × 1 was sampled in the Brillouin zones (Table S1), and the vacuum space was set to 15 Å.

To estimate the ORR catalytic performance of these X@CrS_2_ materials, the change in the Gibbs free-energy change (ΔG) of each elementary reaction step during ORR was computed using the computational hydrogen electrode (CHE) model [15,49]: ΔG = ΔE + ΔZPE − TΔS + ΔG_U_, in which ΔE is the reaction energy directly obtained from DFT computations, and ΔZPE and ΔS represent the difference of zero-point energy and entropy, respectively, which can be derived from the computations on the vibrational frequencies and the standard thermodynamic data. ΔG_U_ = −eU, where U is the applied potential. Since DFT generally fails to obtain the energy of the O_2_ molecule, the free energy of O_2_ (G_O2_) will be computed via the energies of H_2_O and H_2_, namely G_O2_ = G_H2O_ − 2G_H2_ + 4.92 eV. Furthermore, the ORR’s catalytic activity was evaluated by computing the corresponding overpotential (η) according to the following equations: η = max{ΔG_1_, ΔG_2_, ΔG_3_, ΔG_4_}/*e* + U^0^, where ΔG represents the free energy change in each elementary reaction, including the formation of OOH^*^, O^*^, and OH^*^ and the desorption of OH^*^, and U^0^ is the equilibrium potential of ORR, which is equal to 1.23 V. According to the above definition, a smaller η value corresponds to a higher catalytic activity for ORR.

## 3. Results

### 3.1. Structures, Stabilities, and Properties of X@CrS_2_ Catalysts

**Figure 1:**
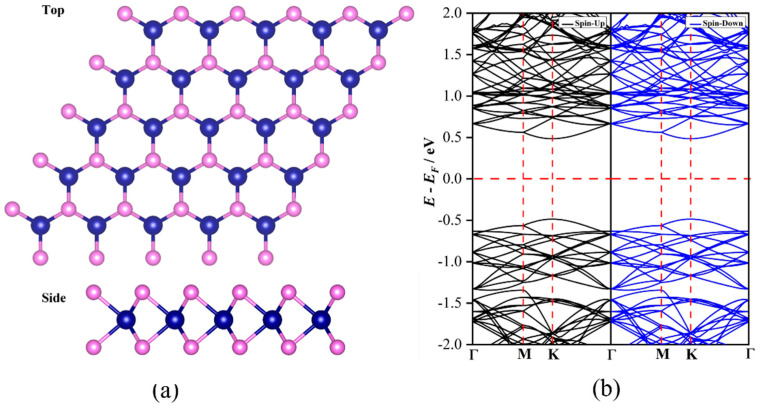
(**a**) The obtained stable configurations and (**b**) computed band structure for the pristine CrS_2_ monolayer.

First, we investigated the structures and electronic properties of the pristine CrS_2_ monolayer with the 2H phase. As shown in Figure 1a, each Cr center is prismatically coordinated by six surrounding S atoms, with the S atoms in the upper layer lying directly above those of the lower layer. Furthermore, the optimized lattice constant for the CrS_2_ monolayer is 3.04 Å, while the lengths of the formed Cr-S bonds are 2.28 Å. Moreover, the CrS_2_ monolayer is a direct semiconductor with the band gap of 0.93 eV, in which the conduction band minimum and the valence band maximum located at the K point mainly originate from the contribution of 3d orbitals of the Cr atom (Figure 1b). Notably, these above results on the structure and properties of a pristine CrS_2_ monolayer are consistent with previous theoretical reports [50], indicating the accuracy of our computational methods to describe the behavior of the CrS_2_ monolayer. 

**Figure 2:**
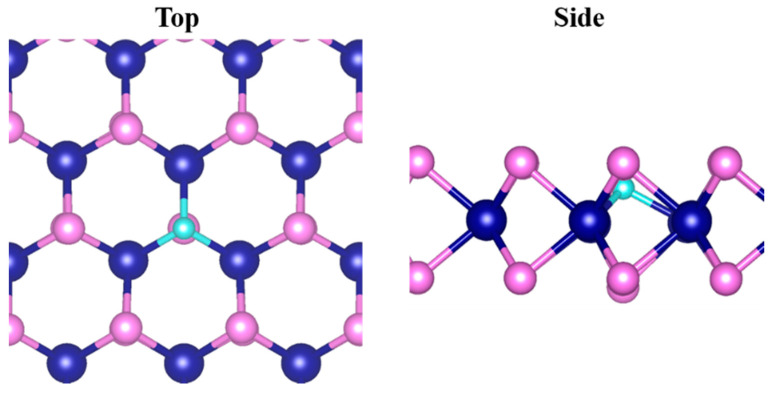
The obtained stable configurations for X@CrS_2_ monolayers (N@CrS_2_ was chosen as a representative).

Based on the optimized CrS_2_ monolayer, various non-metal atoms were introduced to substitute one of the S atoms within the CrS_2_ monolayer to construct the doped CrS_2_ system. After fully geometrical relaxation, the structures of the X@CrS_2_ monolayer are presented in Figure 2, while their corresponding structural parameters are summarized in Table 1. The results showed that, after the introduction of dopants, the structure of the CrS_2_ monolayer is changed in various ways, which are highly dependent on the difference in the radius between dopants and S atoms. In detail, for B, C, N, and O dopants with a smaller radius than the S atom, the introduced non-S atoms are *concave* from the CrS_2_ plane, whereas the doping of Si, P, Cl, As, Se, and Br induces an unchanged or slightly outward structure due to their comparable or slightly larger radius. 

To assess the experimental feasibility of these doped CrS_2_ monolayers, we computed their formation energies (*E_f_*) based on the following equation: *E_f_* = *E*_X@CrS2_ − *E*_CrS2_ + *μ_S_* − *μ_X_* [51,52], where the *E*_X@CrS2_ and *E*_CrS2_ are the total energies of doped and pristine CrS_2_ monolayers, respectively. *μ_X_* and *μ_S_* are the chemical potentials of a doping atom and S atom. Notably, *μ_X_* can be derived from the energy per atom in their bulk X materials. In contrast, *μ_S_* is dependent on the growth conditions, in which two extreme cases were considered, including Cr-rich and S-rich conditions. According to previous studies [32,53], the *μ*_Cr_ and *μ_S_* should meet the relationship from the thermodynamic equilibrium condition: *μ*_CrS2_ = *μ*_Cr_ + *2μ_S_*, where *μ*_CrS2_ represents the total energy of the pristine CrS_2_ monolayer per formula unit. Under Cr-rich conditions, the *μ*_Cr_ was taken from the bulk Cr. Thus, *μ_S_* can be obtained by: *μ*_S_ = (*μ*_CrS2_ − *μ*_Cr_)/2. On the other hand, under S-rich conditions, the *μ_S_* can be obtained from its bulk S_8_ state, and then *μ*_Cr_ can be determined by: *μ*_Cr_ = *μ*_CrS2_ − 2*μ*_S_. The computed formation energies of these X@CrS_2_ materials were listed in Table 1. We can see that the formation energies for B doping are all positive values, indicating that the formation of this kind of doped CrS_2_ monolayer is difficult. On the contrary, when O, Si, Cl, As, Se, and Br dopants were introduced into the CrS_2_ monolayer, their E_f_ values were always negative, implying notable feasibility for their experimental synthesis. Notably, the CrS_2_ monolayers doped by C, N, and P atoms are more likely to form under Cr-rich conditions due to their negative formation energies. Obviously, by carefully tuning the reaction conditions, these doped CrS_2_ monolayers can be easily fabricated in experiments, except for B-doping. 

To further explore the structural stability of X@CrS_2_, we computed the binding energies (*E_bind_*) of the introduced dopants on the CrS_2_ monolayer according to the following equation: *E_bind_ = E*_X@CrS2_ *− E*_CrS2_ *− E*_X_, where *E*_X@CrS2_, *E*_CrS2_, and *E*_X_ represent the total electronic energies of doped CrS_2_ monolayers, the defective CrS_2_ substrate, and isolated X atoms in their most stable phase. It can be observed that the *E_bind_* values of these dopants on the CrS_2_ substrate range from −2.52 eV of Br to −7.16 eV of C, indicative of the strong binding strength, thus ensuring their high stability. The stability of X@CrS_2_ was further evaluated by using AIMD simulations, where N doping was taken as a representative. As shown from Figure S1, there is still no significant distortion of the geometric structure at 500 K, indicative of its high thermodynamic stability. It should be noted that incorporation of various non-metal atoms into 2D TMDs has been already realized experimentally. For example, Li et al. reported a simple, facile, and effective strategy to fabricate an N-doped MoS_2_ nanosheet [54], while Xin et al. demonstrated the fabrication of a P-doped MoS_2_ nanosheet by the one-step hydrothermal method [55]. Therefore, we strongly believe that the as-designed doped CrS_2_ monolayer holds great promise for synthesis.

Next, we explored the magnetic and electronic properties of these X@CrS_2_ systems. Our results showed that the pristine CrS_2_ monolayer is a nonmagnetic material. After introducing these dopants into the CrS_2_ monolayer, however, different magnetic behaviors can be observed: (1) C-, O-, and Se-doped CrS_2_ systems still retain their nonmagnetic states due to the absence of unpaired electrons; (2) as for B, N, P, As, or Br-doped systems, the total magnetic moment is close to 1.00 *μ_B_*, while the Si-doped CrS_2_ monolayer has a magnetic moment of 2.00 *μ_B_*. Moreover, we noted that these dopants will loot different amounts of electrons (0.04~0.95 |e^−^|) from the CrS_2_ monolayer, except for the Si dopant, which can donate about 0.32 electrons to the CrS_2_ substrate. As a result, the band gaps of the doped CrS_2_ monolayer are reduced to different degrees due to the introduction of impurity levels. For example, the N-doped CrS_2_ system exhibits a smaller band gap of 0.25 eV than that of the pristine one (0.99 eV), suggesting the enhanced electrical conductivity, which may facilitate the rapid charge transfer in electrocatalysis [56]. It is noted that the GGA method usually underestimates band gaps, whereas the hybrid functional method, such as Heyd–Scuseria–Ernzerhof (HSE06), can rectify the band gap [57]. For example, the computed band gaps of the well-established MoS_2_ monolayer using HSE06 and PBE methods are 2.20 and 1.79 eV [58], respectively. Despite the fact that a more accurate band gap value can be predicted by means of the HSE06 method, the corresponding computational cost is extremely high for the 10 doped CrS_2_ monolayers, which consist of 25 Cr and 50 S atoms. We will mainly focus on the trend of the band gaps of the CrS_2_ monolayer after non-metal doping.

### 3.2. ORR Catalytic Activity

After knowing that these non-metal doped CrS_2_ candidates exhibit great potential for experimental synthesis, high stability, diverse magnetic moment, and enhanced conductivity, we further explored their catalytic activities towards ORR.

**Figure 3:**
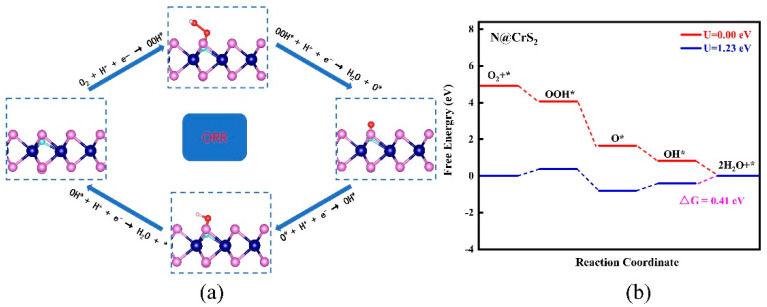
(**a**) The involved reaction pathway and (**b**) the computed free energy diagrams for ORR pathways on the N@CrS_2_ monolayer at zero and applied voltages.

Typically, the O_2_ molecule can be reduced to H_2_O along a four-electron (4e^−^) pathway (Figure 3a): (1) ^*^ + O_2_ (*g*) + H^+^ + e^−^ ⭢ OOH^*^; (2) OOH^*^ + H^+^ + e^−^ ⭢ O^*^ + H_2_O; (3) O^*^ + H^+^ + e^−^ ⭢ OH^*^; (4) OH^*^ + H^+^ + e^−^ ⭢ H_2_O (*l*) [59,60]. We again took the N@CrS_2_ monolayer as the representative to compute the ∆G values of the above four elementary steps. As shown in Figure 3b, we found that the formation of OOH^*^ species on the N site of the N@CrS_2_ catalyst is exothermic, with a ∆G value of −0.84 eV. The length of the formed N–O bond is 1.41 Å, and the O–O bond is elongated to 1.48 Å as compared with that of the free O_2_ molecule (1.23 Å). Subsequently, the approach of a second hydrogen induces the dissociation of OOH^*^ into (O^*^ + H_2_O) or the formation of H_2_O_2_. Remarkably, the OOH^*^ formation is exothermic by 2.43 eV, which is much larger than that of H_2_O_2_ formation (0.16 eV), suggesting that N@CrS_2_ shows a rather high selectivity towards the 4e^−^ pathway by greatly suppressing the competing 2e^−^ one. Once the O^*^ species is formed, it can be further hydrogenated to form OH^*^ and the second H_2_O with the ∆G values of −0.83 and −0.82 eV, respectively. According to the CHE model, the final step (i.e., OH^*^ desorption) is identified as the potential-determining step (PDS). Thus, the limiting potential is 0.82 V, which is the smallest applied voltage to make the whole reaction still exergonic, corresponding to the overpotential of 0.41 V. 

As for other X@CrS_2_ candidates, the computed free adsorption energies of oxygenated species and the corresponding η^ORR^ are summarized in Table S2. We found that the computed η^ORR^ increases in the order of As@CrS_2_ (0.97 V) < C@CrS_2_ (1.20 V) < O@CrS_2_ (1.53 V) < Se@CrS_2_ (1.61 V) < Br@CrS_2_ (1.74 V) ≈ Cl@CrS_2_ (1.78 V) < P@CrS_2_ (2.18 V) < B@CrS_2_ (2.42 V) < Si@CrS_2_ (2.82 V), as shown in Figure S2, which are all higher than that of N@CrS_2_ (0.41 V). In particular, the overpotential of N@VS_2_ (0.41 V) is even lower than that of the well-established Pt catalyst (0.45 V) [15], implying its excellent ORR catalytic activity. As ORR usually occurs in aqueous solution, we also studied the solvent effect on the ORR activity of the N@CrS_2_ catalyst using the implicit solvation model in VASPsol with a dielectric constant of 80 [61]. It can be seen from Figure S3 that the PDS locates at the last step, and the computed η value is 0.48 V, which is comparable to that of the value without the solvent effect (0.41 V), implying that the solvent effects left the superior ORR catalytic performance of the N@CrS_2_ monolayer nearly unchanged. Although implicit solvent models can offer fast and inexpensive starting points for estimating the solvation effects [62,63,64,65], they may not fully describe the interactions of ORR adsorbates with water molecules due to the formation of H-bonds. As an alternative to implicit solvent approaches, explicit solvent models may provide a more comprehensive solution to describe the solvation effects on the ORR catalytic performance, which usually requires sampling thousands of solvent configurations, thus resulting in significant computational expense due to the use of classical molecular dynamic (MD) computations based on force fields. To this end, according to previous studies [66], explicit water layers were employed by placing 10 H_2_O molecules on the adsorbed oxygenated species (Figure S4). The results showed that the computed η value for ORR on the N@CrS_2_ catalyst using the explicit models is 0.50 V (Figure S4), which is close to our implicit solvent value (0.48 V). Thus, the implicit solvation model can also provide a reasonable estimation of the solvation energy for ORR intermediates, consistent with previous theoretical studies [67]. 

## 4. Discussion

Based on the well-accepted Sabatier principle, either too strong or too weak adsorption of reaction intermediates on catalysts can result in poor catalytic activity. This is because adsorption that is too strong will hamper the desorption process, resulting in poisoned catalysts, whereas too weak adsorption will induce insufficient activation of intermediates. Thus, the best catalysts exhibit the optimal adsorption strength with reaction intermediates, which locates at the peak of the volcano plot. Clearly, the ORR’s catalytic activity is intrinsically dependent on the adsorption strength of reaction intermediates with catalysts. To this end, we scaled the adsorption free energies of OOH^*^ (ΔG_OOH*_), O^*^ (ΔG_O*_) and OH^*^ (ΔG_OH*_) on different X@CrS_2_ systems. 

**Figure 4:**
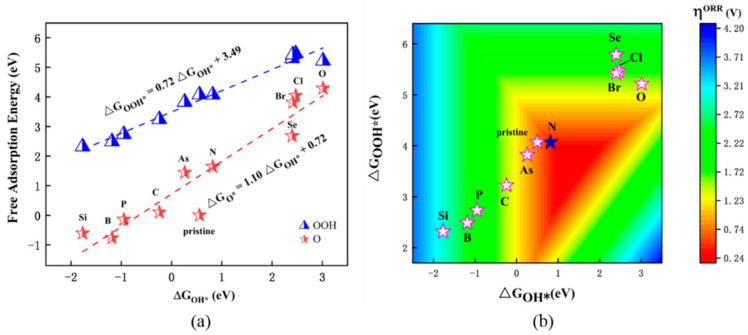
(**a**) Scaling relations between the free adsorption energies of intermediates (ΔG_O*_ vs. ΔG_OH*_ and ΔG_OOH*_ vs. ΔG_OH*_) and (**b**) η^ORR^ vs. ΔG_OH*_ on different X@CrS_2_ systems.

As shown in Figure 4a, obvious linear scaling relationships can be obtained between ΔG_OOH*_ and ΔG_OH*_ by ΔG_OOH*_ = 0.72 ΔG_OH*_ + 3.49 (R^2^ = 0.97) as well as ΔG_O*_ and ΔG_OH*_ by ΔG_O*_ = 1.10 ΔG_OH*_ + 0.72 (R^2^ = 0.89). Thus, ΔG_OH*_ can be utilized as an eligible descriptor to describe the catalytic trend of these considered X@CrS_2_ catalysts. Furthermore, a volcano plot of ORR activity (η^ORR^) for X@CrS_2_ with the variation in ΔG_OH*_ is obtained (Figure 4b), in which either a strong Si@CrS_2_ or a weak binding strength O@CrS_2_ with OH^*^ species will induce poor ORR activity. On the contrary, the N@CrS_2_ catalyst displays a moderate binding strength with OH^*^ and exhibits high ORR catalytic activity, making it locate at the peak of the volcano curve, and, thus, it becomes the best ORR catalyst among all the studied systems. 

**Figure 5:**
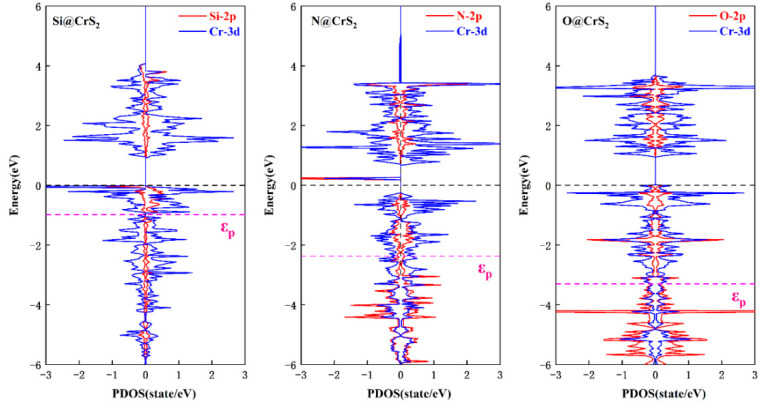
The computed partial density of states for Si@CrS_2_, N@CrS_2_, and O@CrS_2_. The black and pink vertical dotted lines represent the Fermi energy level and p-band center (ε_p_), respectively.

To gain deep insight into the remarkable difference of OH^*^ adsorption on X@CrS_2_, we turn to exploring the p-band center (ε_p_) model of the non-metal active sites [68], in which Si@CrS_2_, N@CrS_2_, and O@CrS_2_ systems were chosen as the representatives of strong, moderate, and weak OH^*^ adsorption. Notably, according to this model, the position of ε_p_ closer to the Fermi level will generally induce a stronger interaction of reaction species with catalysts. Our results demonstrated that the computed ε_p_ values of Si, N, and O dopants are −0.98, −2.37, and −3.30 eV, respectively, as shown in Figure 5. The moderate ε_p_ value on N@CrS_2_ suggests its optimal interaction with the oxygenated species, which is responsible for its superior catalytic performance. 

**Figure 6:**
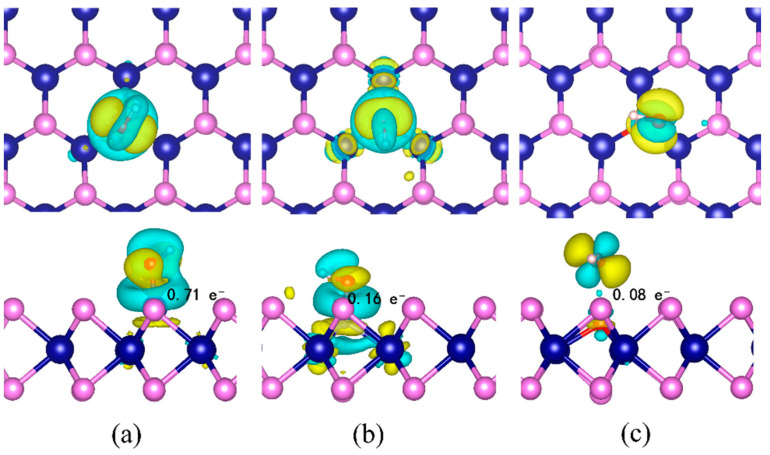
The charge difference density of OH^*^ adsorption on (**a**) Si@CrS_2_N, (**b**) N@CrS_2_, and (**c**) O@CrS_2_ with the isosurface of 0.003 e/Å^3^.Cyane and yellow bubbles represent positive and negative charges, respectively.

In addition, the charge density differences in Si@CrS_2_, N@CrS_2_, and O@CrS_2_ with adsorbed OH^*^ species were computed (Figure 6). Upon OH^*^ adsorption, we found that the charge depletion around the Si, N, and O endures, while the charge accumulation locates at the X–O bonds, indicating the charge transfer from the catalysts to oxygenated intermediates. According to the Bader charges analysis, the charge transfer is 0.71, 0.08, and 0.16 |e^−^| for OH^*^ adsorption on Si@CrS_2_, N@CrS_2_, and O@CrS_2_, respectively, which is consistent with the binding strengths between them. Thus, the moderate adsorption strength of N@CrS_2_ endows it with its high ORR catalytic activity.

## 5. Conclusions

In summary, by performing comprehensive DFT computations, we have systematically investigated the structures, stabilities, and magnetic and electronic properties as well as the ORR catalytic activity of several non-metal doped CrS_2_ monolayers. Our results demonstrated that most of the doped CrS_2_ materials generally possess high stability and hold great promise for experimental synthesis. As expected, depending on the kinds of the incorporated dopants, these CrS_2_ monolayers exhibit different magnetic and electronic properties. Based on the computed free energy changes in all elementary steps during ORR, the N@CrS_2_ was revealed as a quite promising ORR electrocatalyst due to its lower overpotential (0.41 V) than that of the Pt benchmark (0.45 V). Moreover, obvious scaling linear relationships between oxygenated species can be obtained, which was employed to construct the volcano curve between ORR catalytic activity and OH^*^ binding strength. Understandably, the moderate p-band center and charge transfer from catalyst to oxygenated species render the N@CrS_2_ catalyst’s optimal interaction with reaction species, thus rationalizing its outstanding ORR catalytic performance. Our findings not only provide a novel strategy for the design of low-cost, highly-efficient ORR electrocatalysts, but also further widen the potential applications of CrS_2_-based materials.

## Figures and Tables

**Table 1:** The computed bond’s length of X-Cr (d_X-Cr_, Å), formation energies (*E_f_,* eV), binding energies (*E_bind_,* eV), magnetic moment (*μ_B_*), charge transfer (Q, e^−^), and band gaps (*E_gap_*, eV) for various X@CrS_2_ monolayers.

	d_X-Cr_	*E_f_*		*E_bind_*	*μ_B_*	Q	*E_gap_*
		S-rich	Cr-rich				
pristine	2.28	/	/	/	0.00	/	0.93
B	1.97	2.06	0.94	−5.62	1.00	0.09	0.20
C	1.92	0.76	−0.36	−7.16	0.01	0.77	0.90
N	1.87	0.16	−0.96	−5.75	1.00	0.94	0.25
O	1.93	−2.70	−3.83	−6.96	0.01	0.95	0.96
Si	2.47	−3.40	−4.53	−3.93	2.00	0.32	0.27
P	2.35	0.15	−0.97	−4.15	1.00	0.27	0.95
Cl	2.41	−2.48	−3.61	−3.14	1.00	0.47	0.18
As	2.49	−0.02	−1.14	−3.70	0.96	0.04	0.00
Se	2.42	−1.17	−2.30	−4.76	0.01	0.28	0.98
Br	2.56	−0.46	−1.58	−2.52	1.00	0.31	0.19

## Supplementary Materials

The following supporting information can be downloaded at: www.mdpi.com/article/10.3390/nano12173012/s1, Figure S1: Total potential temperature and energy fluctuations after equilibration for N@CrS_2_ in the AIMD simulation; Figure S2: The computed free energy diagrams for ORR on different X@CrS_2_ systems; Figure S3: The computed free energy diagrams for ORR on N@CrS_2_ with solvent effect. Figure S4: The computed free energy diagrams and the corresponding intermediates configurations for ORR on N@CrS2 with the explicit solvent models; Tables S1: The computed the overpotentials (η^ORR^, V) at various energy cutoff (eV) or k-points; Tables S2: The computed the overpotential (η^ORR^) and the free adsorption energies (ΔG, eV) for various X@CrS2 materials.
