# Supplementary material for: N-Doped CrS2 Monolayer as a Highly-Efficient Catalyst for Oxygen Reduction Reaction: A Computational Study
Source: Nanomaterials (Basel). 2022 Aug 30;12(17):3012. doi: 10.3390/nano12173012 (PMC9458212; doi:10.3390/nano12173012)
Supplement: Supplementary file 1 [file nanomaterials-12-03012-s001.zip › nanomaterials-1845575-supplementary.pdf]

Supplementary Material

N-Doped CrS_2 Monolayer as a Highly-Efficient Catalyst for Oxygen Reduction Reaction: A Computational Study

Zengming Qin [1], Zhongxu Wang [1], Xiaofeng Li [2], Qinghai Cai [2], Fengyu Li [3,*] and Jingxiang Zhao [1,2,*]

[1] Key Laboratory for Photonic and Electronic Bandgap Materials, Ministry of Education, School of Physics and Electronic Engineering, Harbin Normal University, No. 1, Shida Street, Harbin 150025, China
[2] College of Chemistry and Chemical Engineering, Harbin Normal University, Harbin 150025, China
[3] School of Physical Science and Technology, Inner Mongolia University, Hohhot 010021, China
* Correspondence: fengyuli@imu.edu.cn (F.L.); zhaojingxiang@hrbnu.edu.cn (J.Z.)

Table S1. The computed the overpotentials (η^{ORR}, V) at various energy cutoff (eV) or k-points.

cutoff	η^{ORR}	k-point	η^{ORR}
400	0.43	2 × 2 × 1	0.40
450	0.44	3 × 3 × 1	0.41
500	0.41	4 × 4 × 1	0.41
500	0.41	5 × 5 × 1	0.41

Table S2. The computed the overpotential (η^{ORR}) and the free adsorption energies (ΔG, eV) for various X@CrS_2 materials.

	η^{ORR}	ΔG_{O*}	ΔG_{OH*}	ΔG_{OOH*}
pristine	1.78	0.00	0.55	4.08
B	2.42	-0.76	-1.19	2.49
C	1.20	0.13	0.10	3.23
N	0.41	1.65	0.82	4.07
O	1.53	4.29	3.01	5.22
Si	2.82	-0.56	-1.59	2.21
P	2.18	-0.14	-0.95	2.74
Cl	1.78	4.04	2.47	5.47
As	0.97	1.45	0.26	3.83
Se	1.61	2.68	2.40	5.30
Br	1.74	3.82	2.41	5.43

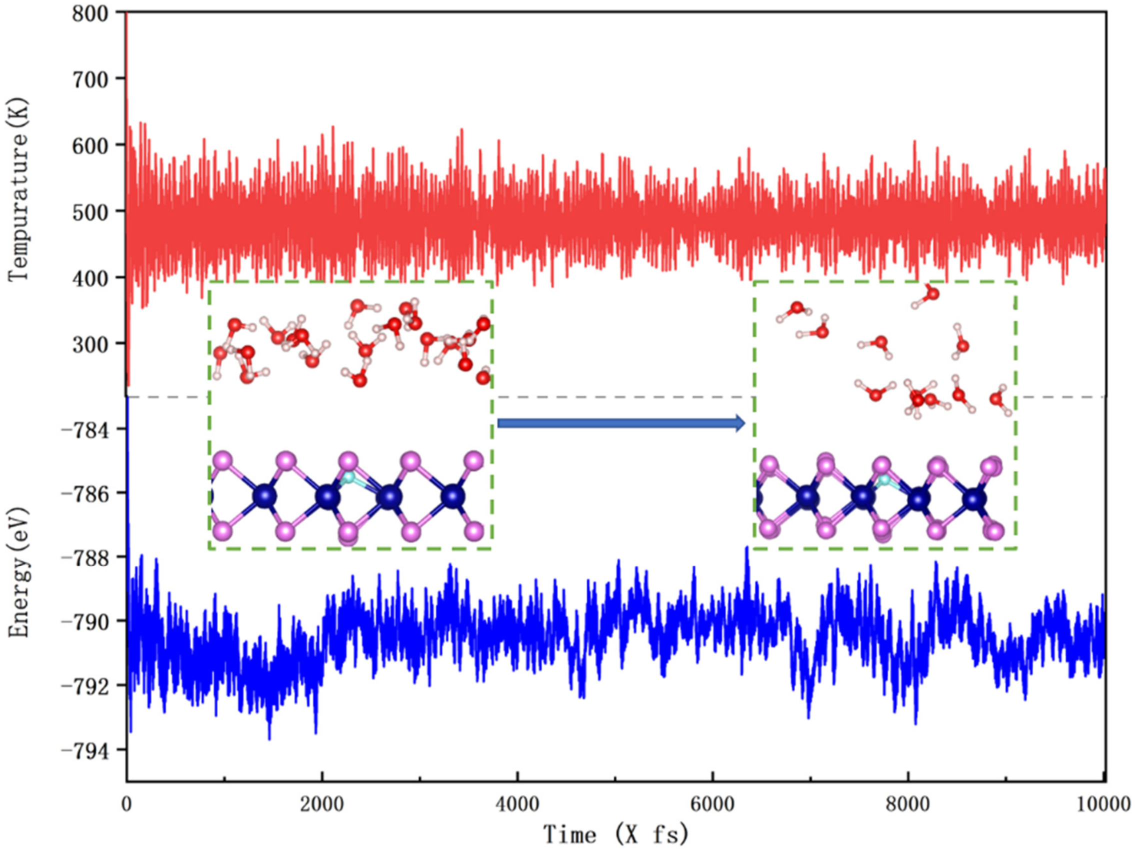

Figure S1. Total potential temperature and energy fluctuations after equilibration for N@CrS_2 in the AIMD simulation.

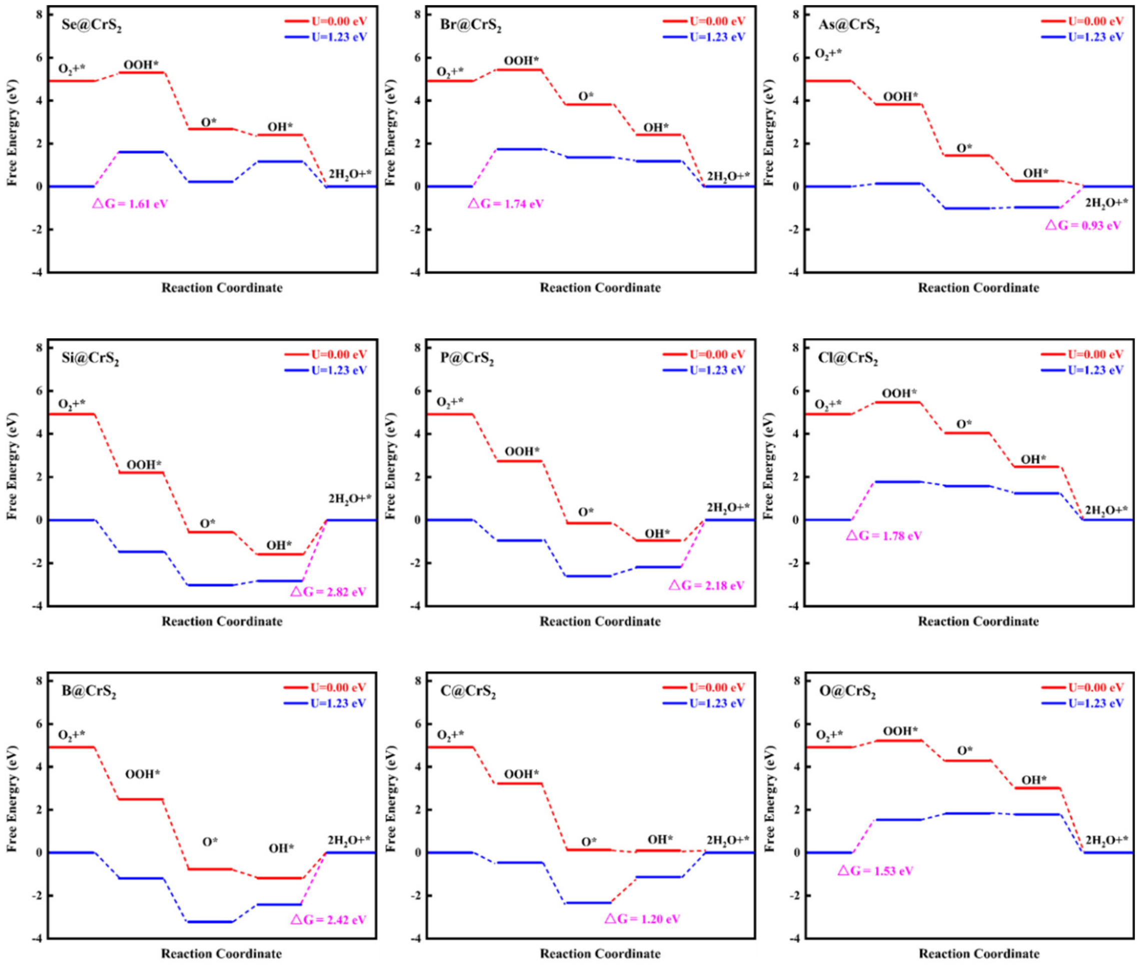

Figure S2. The computed free energy diagrams for ORR on different X@CrS$_2$ systems.

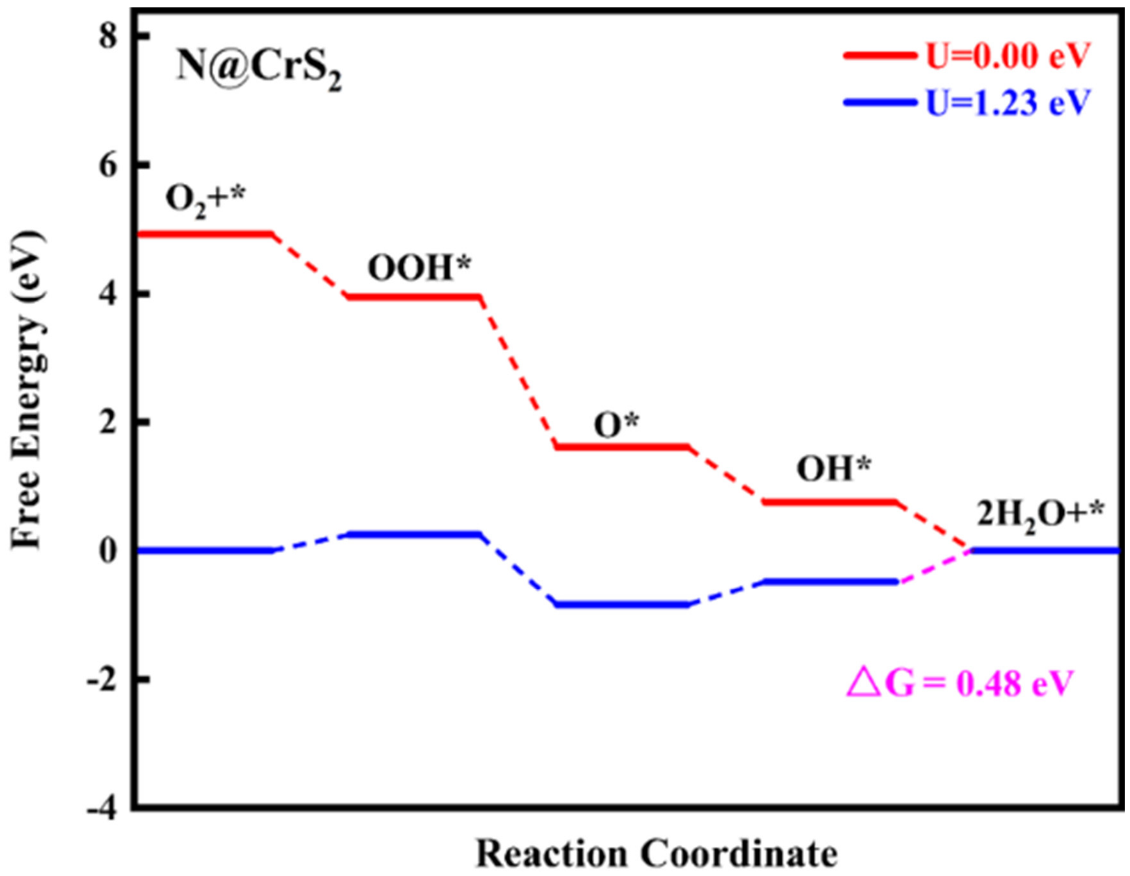

Figure S3. The computed free energy diagrams for ORR on N@CrS2 with solvent effect.

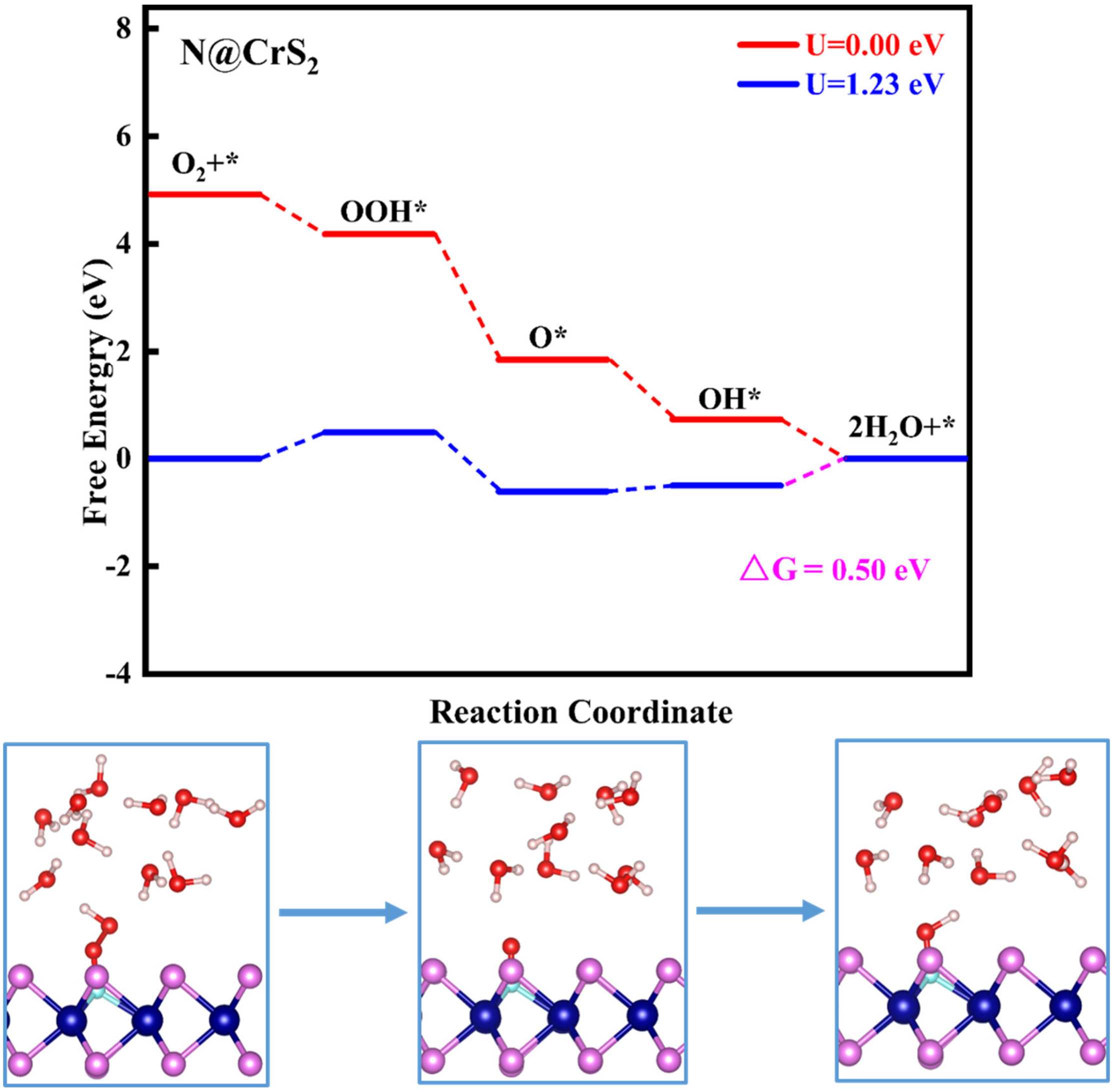

Figure S4. The computed free energy diagrams and the corresponding intermediates configurations for ORR on N@CrS_2 with the explicit solvent models.
